# Effectiveness of psychological therapies for depression and anxiety in atypical dementia

**DOI:** 10.1002/alz.14332

**Published:** 2024-10-23

**Authors:** Celine El Baou, Rob Saunders, Joshua E. J. Buckman, Marcus Richards, Claudia Cooper, Natalie L. Marchant, Roopal Desai, Georgia Bell, Caroline Fearn, Stephen Pilling, Nikki Zimmermann, Val Mansfield, Sebastian Crutch, Emilie Brotherhood, Amber John, Joshua Stott

**Affiliations:** ^1^ Adapt Lab Research Department of Clinical Educational and Health Psychology UCL London London UK; ^2^ Centre for Outcomes Research and Effectiveness Research Department of Clinical Educational and Health Psychology UCL London UK; ^3^ iCope – Camden & Islington NHS Foundation Trust St. Pancras Hospital London UK; ^4^ MRC Unit for Lifelong Health and Ageing at UCL UCL London UK; ^5^ Centre for Psychiatry and Mental Health Wolfson Institute of Population Health Queen Mary University London UK; ^6^ Division of Psychiatry UCL London UK; ^7^ Camden & Islington NHS Foundation Trust St. Pancras Hospital London UK; ^8^ Dementia Research Centre UCL Queen Square Institute of Neurology London UK

**Keywords:** anxiety, atypical dementia, depression, primary care, psychological therapies

## Abstract

**INTRODUCTION:**

People with dementia may benefit from psychological therapies for depression or anxiety, but evidence of their effectiveness in atypical dementia is limited.

**METHODS:**

Using electronic health‐care records of > 2 million people who attended psychological therapy services in England between 2012 and 2019, we examined pre–post therapy symptom changes and compared therapy outcomes among 523 people with atypical dementia, a matched cohort without dementia, and 1157 people with typical dementia.

**RESULTS:**

People with atypical dementia experienced reductions in depression (Cohen *d* = −0.92 [−1.05 to −0.79]) and anxiety (*d* = −0.85 [−0.98 to −0.73]) symptoms. They had similar odds of improvement than people with typical dementia (odds ratio [OR] = 1.07, 95% confidence interval [CI]: 0.85 to 1.34), but lower odds than people living without dementia (OR = 0.70, 95% CI: 0.53 to 0.91). Reasons for discharge were similar between all groups.

**DISCUSSION:**

People with atypical dementia may benefit from primary care psychological therapies, but further research is needed to explore necessary adaptations.

**Highlights:**

- Talking therapies for depression and anxiety may be beneficial for people with atypical dementia.
- Being younger and having a lower socioeconomic background are associated with poorer outcomes.
- Receiving more treatment sessions and shorter waiting times are associated with better outcomes.

## 1 BACKGROUND

Dementia is a leading cause of death worldwide.^1^ Approximately 8% of all dementias do not have either amnestic Alzheimer's disease presentation, or vascular causes^2^ and conditions with less commonly diagnosed presentations (hereafter “atypical dementias”) such as frontotemporal dementia (FTD),^3^, ^4^ dementia with Lewy bodies (DLB),^5^ and posterior cortical atrophy (PCA),^6^ are associated with symptoms that are not memory led, and may primarily affect behavior, language, or visual processing.^7^, ^8^ It is estimated that 39% of people living with dementia experience depression and there is a similar prevalence rate reported for anxiety.^9^ Moreover, the prevalence of depression and anxiety tends to be higher in atypical dementias.^10^, ^11^, ^12^, ^13^, ^14^ Evidence‐based psychological therapies are recommended for the treatment of depression or anxiety in people living with dementia, and previous research has found that such therapies may be effective.^15^ However, research has focused on more typical dementias, and the effectiveness of these therapies in routine care settings is unknown for people living with atypical dementia. Addressing this is crucial because of the specific challenges associated with atypical dementias, which may mean that different therapy adaptations are needed for these dementias (e.g., incorporating communication strategies for language‐led forms of FTD) and that therapy outcomes might be affected by the type of dementia because different symptomatology might affect the ability to use therapeutic strategies. This means that living with atypical dementia may be associated with differences in therapy outcomes and reasons for discharge (with potentially higher dropout rates) compared to people living with typical dementia and no dementia. Therefore, this study included the following four goals:

Aim 1. Examine whether people with atypical dementias experienced a reduction in their symptoms of depression or anxiety after receiving psychological therapy in a general primary care setting for adults with depression or anxiety.
Aim 2. Identify pretreatment factors associated with any such improvement.
Aim 3. Compare therapy outcomes for people living with atypical dementia versus typical dementia, and versus no dementia.
Aim 4. Compare reasons for discharge from services between people with atypical dementia versus typical dementia, and versus no dementia.

## 2 METHODS

### 2.1 Data sources and procedures

The MODIFY study^15^, ^16^, ^17^ includes linked data across all services and health regions in England included in the National Health Service Talking Therapies for anxiety and depression (NHS TTad) database,^18^ the Hospital Episode Statistics (HES) database,^19^ the linked HES–Office for National Statistics (ONS) mortality dataset,^20^ and the Mental Health Services Data Set^21^ (see Appendix S1 in supporting information for more details). Records available from database inception (in 2012) up to the latest data point (2019) available at time of analysis were pseudonymized and linked using a key provided by NHS Digital; as such, informed consent was not required for this study. This work uses data provided by patients and collected by the NHS as part of their care and support.Departmental ethical approval was granted by the Division of Psychology and Language Sciences at University College London. NHS TTad can be accessed by anyone in England through referral from any health or social care practitioner. People can self‐refer by contacting their local NHS TTad service, or referral can also be made by a care partner on behalf of the person trying to access therapy. People may then receive a course of therapy, or be discharged or sign‐posted to other services after clinician assessment by an NHS TTad practitioner. For those people who receive a course of treatment, psychological therapies are provided according to a stepped‐care model in accordance with clinical guidelines.^22^ People with less complex challenges are commonly offered short‐term (≤ 8 sessions) “low‐intensity” guided self‐help therapies, which may be delivered in person or online, in one‐to‐one or group settings. When people do not respond to low‐intensity therapy or their challenges are more complex at referral, individuals are commonly offered high‐intensity therapies (≥ 16–20 sessions), which are formulation driven one‐to‐one cognitive behavioral or other types of evidence‐based therapies. Interventions are standardized and delivered by trained practitioners and psychotherapists following evidence‐based protocols (see Appendix S2 in supporting information for more details). Analyses were reported following Reporting of Studies Conducted Using Observational Routinely Collected Data^23^ (RECORD) principles (see Appendix S3 in supporting information for a checklist).

**RESEARCH IN CONTEXT**

1. **Systematic review**: The authors reviewed the literature using traditional (e.g., PubMed) research databases, and found indications that evidence‐based psychological therapies may benefit people living with the most common types of dementia and that specific subtypes, such as frontotemporal dementia, may be associated with poorer therapy outcomes. Such references are cited in the text. No study investigated outcomes and predictors of outcomes of psychological therapies provided in routine care settings in a cohort of people living with atypical dementia.
2. **Interpretation**: Our findings suggest that people living with atypical dementia are as likely to benefit from therapy as people with typical dementia types and that being older (age > 65), receiving more treatment sessions, shorter waiting times, and higher socioeconomic status were associated with better therapy outcomes.
3. **Future directions**: Future research should explore how to optimize therapies for people living with atypical dementia, how access to health services may be improved, and how specific dementia symptoms and experiences may affect therapy outcomes.

### 2.2 Study population

The study cohort included people referred to NHS TTad aged ≥ 18 who met a set of criteria used in previous research^15^, ^24^:

  1. Met clinical “caseness” thresholds (a level of symptoms likely to be sufficient for meeting diagnostic criteria for the measured condition) for depression or anxiety measured using the Patient Health Questionnaire^25^ (PHQ‐9) and the Generalized Anxiety Disorder questionnaire^26^ (GAD‐7; for further details see section 2.4);
  2. Were ascertained to have an atypical dementia diagnosis, a typical dementia diagnosis, or no dementia diagnosis as defined in section 2.3, and could consequently be assigned to one of the three groups examined here;
  3. Received at least one course of treatment within NHS TTad (attending at least two NHS TTad treatment sessions and having been discharged from the episode of care); when individuals received several courses of treatment, the first course was included in analyses.

### 2.3 Dementia ascertainment

Atypical dementia code ascertainment was carried out based on previous research: code lists based on the International Classification of Diseases 10th revision (ICD‐10) were extracted from those studies included in a review of rare and atypical dementias prevalence^27^ identifying these dementias using the ICD coding system. The extracted codes were independently reviewed by three experts in atypical dementia research (J.S., S.C., E.B.), and a consensus was reached in a resolution meeting for any discrepancy. (See Appendix S4 in supporting information for the final code list.) In addition, a review of ICD‐10 codes not previously used in research but potentially associated with atypical dementia by expert clinician researchers (S.C., J.S.) led to the identification of ICD‐10 code G31.9 (Degenerative disease of nervous system, unspecified) as potentially including many people living with atypical dementia. The impact of using this code in addition to previously identified ICD‐10 code was evaluated in sensitivity analyses.

Atypical dementia was defined as having at least one atypical dementia diagnosis code before referral to NHS TTad. Two comparison groups (typical dementia and no dementia) were used in the analyses. Typical dementia was defined as having a dementia diagnosis (codes based on previous research,^28^ see Appendix S4 for list) before referral to NHS TTad, and no atypical dementia diagnosis at any point in their record. People were included in the “no dementia” group if no diagnosis of typical or atypical dementia was found in their record before, during, or at any time after NHS TTad treatment.

### 2.4 Outcomes

Depression and anxiety symptoms were evaluated before and at the last therapy session, using routine NHS TTad services measures. Depression was assessed using the PHQ‐9 (caseness threshold score of ≥10). The PHQ‐9 was previously found helpful in screening for depression in primary care memory clinics.^29^ Generalized anxiety was assessed using the GAD‐7 (caseness threshold score of ≥ 8^26^).

To examine whether people living with atypical dementias experienced a reduction in symptoms (Aim 1) the outcome measure was the absolute change in symptoms of anxiety and the absolute change in symptoms of depression from first to last attended therapy session in NHS TTad services. To evaluate associations between pretreatment characteristics and improvement in symptoms (Aim 2), and to compare outcomes among those with atypical dementias versus other dementias versus no dementia, the outcome was a “reliable improvement*.”* This is an empirically based metric^18^ used in NHS TTad services that combines measures of depression and anxiety to define a statistically reliable improvement in symptoms between the first and last treatment session (≥ 6 points on PHQ‐9, ≥ 4 points on GAD‐7). For between‐group comparison of reasons for discharge (Aim 4), the outcome was the reason for discharge from therapy coded by the treating clinician based on a set of codes used in all NHS TTad services (“treatment completed,” “referred on to specialist care,” “dropped‐out/premature end of therapy,” “declined care,” and “service not suitable”).

### 2.5 Covariates

Analyses included age, sex, ethnicity, Index of Multiple Deprivation (IMD) quintile (an official measure of relative deprivation in England that ranks small geographical areas from the most deprived to the least deprived), self‐reported psychotropic medication use, presence of a long‐term health condition, year of appointment, baseline anxiety and depression scores, number of treatment sessions, and waiting times as covariates (see Appendix S5 in supporting information for more details). To account for regional and service effects, a measure of the NHS organization planning resources in specific regional areas (integrated care board [ICB]) was also included in the propensity score matching algorithm.

### 2.6 Statistical analyses

To meet Aims 1 and 2, analyses were conducted on the cohort of people with atypical dementia diagnoses. The mean changes in PHQ‐9 and GAD‐7 scores pre–post treatment were analyzed using *t* tests (Aim 1). Multivariable logistic regression models were fitted to identify associations between pretreatment characteristics and reliable improvement (Aim 2).

A second set of analyses was conducted (Aim 3 and 4), using separate models with either typical dementia or no dementia as a comparison group.

First, we explored whether having an atypical dementia diagnosis (vs. typical dementia or no dementia diagnosis) is associated with a lower likelihood of reaching reliable improvement after a course of psychological therapy. Logistic regression models were run in the following sequence: Model 1 included group (atypical dementia vs. typical dementia or no dementia) as a covariate; Model 2 additionally adjusted for covariates previously described. When the comparison group was the “no dementia group,” Model 3 was rerun using propensity score^30^–matched samples.

For these matched analyses, a propensity score estimating the probability of having an atypical dementia diagnosis (vs. no dementia diagnosis) was estimated using logistic regression including all factors possibly associated with the outcomes as covariates (as listed in section 2.5). Exact matching was used for NHS TTad service (see Appendix S6 in supporting information for more details). Nearest neighbor matching was used, as well as a calliper set to 0.1 for 1:1 propensity score matching. When a control was identified as an appropriate match for more than one participant, these were weighted and used in the analysis, allowing a control to be matched to a maximum of two individuals. Robust standard errors were used in the matched analyses to account for the fact that the propensity score was estimated. The propensity score–matched model was considered the primary model. Matched analyses were not conducted for the atypical dementia versus typical dementia analyses due to insufficient sample sizes. For these analyses, the adjusted model was considered the primary model.

We then used similar methods to explore whether having an atypical dementia diagnosis versus typical dementia or no dementia diagnosis is associated with different discharge reasons at the end of treatment. Multilevel multinomial logistic regression was used, adjusted sequentially by different sets of covariates as in Models 1–3 described previously.

### 2.7 Missing data

For categorical covariates, a “missing” data category was dummy coded. For the sex variable, missing records were excluded; the missing category represented 0.003% of the overall study cohort and as such a small proportion of missing data was unlikely to affect the analyses. No data were missing for continuous covariates.

### 2.8 Supplementary analyses

To provide more context to the findings, supplementary analyses were run considering two additional outcomes for Aim 2 and Aim 3: “reliable recovery” and “reliable deterioration” as outcomes for Aim 2 and Aim 3. In these analyses, reliable recovery was defined as meeting reliable improvement and additionally ending treatment before the caseness threshold for both depression and anxiety. This measure was used to identify which proportion of people living with dementia have a stronger response to therapy. Reliable deterioration was defined as a clinically meaningful increase in symptoms between the first and last treatment session (≥ 6 points on PHQ‐9, ≥ 4 points on GAD‐7). This measure was used to identify which proportion of people living with dementia may be at higher risk of deteriorating mental health after a course of therapy.

### 2.9 Sensitivity analyses

As described previously, the impact of using a broader range of diagnosis codes for atypical dementia ascertainment was evaluated by rerunning analyses including code G31.9 in the atypical dementia group.

### 2.10 Patient public involvement

Two patient public involvement (PPI) representatives (N.M. and V.M.) were included in this project and attended regular meetings over the conduct of the study. They confirmed the aims of the project fit with PPI priorities and will be involved in codesigning materials to disseminate the findings of this research to the wider public.[Table alz14332-tbl-0001]

## 3 RESULTS

### 3.1 Demographic and sample characteristics

Among the 2,292,968 individuals who received at least two treatment sessions in NHS TTad, 573 individuals with an atypical dementia diagnosis before referral were identified, along with 1157 individuals with a typical dementia diagnosis, and 1,880,357 with no diagnosis of dementia; 410,881 individuals were not included in the analyses because they did not meet caseness thresholds for either depression and anxiety (*N* = 268,417), had missing or incomplete depression or anxiety measures at baseline (*N* = 112,710), a diagnosis code of any dementia after therapy (*N* = 13,435), missing sex information (*N* = 6986), or a suspected recording error (such as date of referral after date of assessment; *N* = 9333).

Demographic and sample characteristics before and after matching are included in Table 1. Compared to people with typical dementia, people with atypical dementia were younger, less likely to live in the most socially deprived areas, more likely to experience depression as their primary diagnosis than anxiety, and more likely to receive more treatment sessions. Compared to people without dementia, people with atypical dementia were older, more likely to be male, to take psychotropic medications, to report long‐term health conditions, and to experience depression as their primary diagnosis.

**TABLE 1 alz14332-tbl-0001:** Demographics, baseline characteristics, and outcomes.

Demographic and baseline measures	Atypical dementia diagnosis *N* = 523	Typical dementia diagnosis *N* = 1157	No dementia diagnosis *N* = 1 880 357	No dementia diagnosis—matched sample *N* = 521
Age at referral—Mean (SD)	61.0 (17.05)	65.2 (16.4)	40.4 (14.7)	58.44 (16.21)
	***n* (%)**	***n* (%)**	***n* (%)**	***n* (%)**
**Age category**				
<65	263 (50.28%)	496 (42.83%)	1 758 985 (93.55%)	263 (50.5%)
65+	260 (49.71%)	661 (57.08%)	121 372 (6.45%)	258 (49.52%)
**Ethnicity**				
White	430 (82.22%)	911 (78.74%)	1 538 900 (81.84%)	429 (82.34%)
Mixed	(0.8%)*	13 (1.12%)	36 592 (1.95%)	(0.8)*
Asian	11 (2.10%)	57 (4.93%)	77 935 (4.14%)	11 (2.11%)
Black	11 (2.10%)	48 (4.15%)	47 259 (2.51%)	11 (2.11%)
Chinese	5 (0.96%)	(0.1%)*	3 625 (0.19%)	0 (0.00%)
Other	0 (0.0%)	18 (1.56%)	20 500 (1.09%)	5 (0.96%)
Missing	62 (11.85%)	109 (9.42%)	155 546 (8.27%)	61 (11.71%)
**Sex**				
Male	223 (42.64%)	478 (41.31%)	619 302 (32.94%)	221 (42.42%)
Female	300 (57.36%)	679 (58.69%)	1 261 055 (67.06%)	300 (57.58%)
**Index of Multiple Deprivation (quintile)**				
1 (Most deprived)	114 (21.80%)	328 (28.35%)	406 994 (21.64%)	113 (21.69%)
2	105 (20.08%)	255 (22.04%)	402 657 (21.41%)	105 (20.15%)
3	117 (22.37%)	225 (19.45%)	367 027 (19.52%)	117 (22.46%)
4	97 (18.55%)	174 (15.04%)	336 082 (17.87%)	96 (18.43%)
5 (Least deprived)	73 (13.96%)	145 (12.53%)	304 664 (16.20%)	73 (14.01%)
Missing	17 (3.25%)	30 (2.59%)	62 935 (3.35%)	17 (3.26%)
**Taking psychotropic medication before therapy**				
No	173 (33.08%)	337 (29.13%)	813 935 (43.29%)	172 (33.01%)
Yes	295 (56.41%)	613 (52.98%)	890 771 (47.37%)	295 (56.62%)
Missing	55 (10.52%)	207 (17.89%)	175 651 (9.34%)	54 (10.36%)
**Self‐reported long‐term health condition**				
No	130 (24.86%)	292 (25.24%)	1 051 024 (55.89%)	130 (24.95%)
Yes	290 (55.45%)	577 (49.87%)	433 415 (23.05%)	288 (55.28%)
Missing	103 (19.69%)	288 (24.89%)	395 918 (21.06%)	103 (19.77%)
**Diagnosis category**				
Depression	219 (41.87%)	383 (33.10%)	561 339 (29.85%)	219 (42.03%)
Mixed anxiety and depressive disorder	73 (13.96%)	166 (14.35%)	320 618 (17.05%)	73 (14.01%)
GAD	60 (11.47%)	140 (12.10%)	288 696 (15.35%)	60 (11.52%)
OCD	(0.4%)*	(0.2%)*	22 390 (1.19%)	2 (0.38%)
PTSD	8 (1.53%)	19 (1.64%)	38 811 (2.06%)	8 (1.54%)
Phobic anxiety and panic disorder	21 (4.02%)	36 (3.11%)	105 617 (5.62%)	21 (4.03%)
Unspecified anxiety	0 (0.0%)	(0.4%)*	6 109 (0.32%)	0 (0.0%)
Missing	140 (26.77%)	407 (35.18%)	536 777 (28.55%)	138 (26.49%)
**Waiting times**				
Weeks between ref. and assessment	3.04 (4.10) 0–28	3.11 (4.01) 0–28	3.24 (4.36) 0–28	2.98 (4.08) 2–23
% > 6 weeks	67 (12.81%)	161 (13.92%)	160 213 (13.53%)	67 (12.86%)
Weeks between assessment and treatment	10.05 (11.11) 0–36	10.42 (11.69) 0–36	9.30 (10.82) 0–36	9.64 (10.8) 0–36
% > 18 weeks	121 (23.14%)	289 (24.98%)	417 779 (21.70%)	121 (23.22%)
**Number of treatment sessions**				
Number of sessions—mean (SD)	5.9 (3.98)	5.35 (3.78)	6.3 (4.27)	6.2 (4.29)
≥5 low‐intensity sessions	83 (15.87%)	167 (14.43%)	338 637 (18.01%)	69 (13.24%)
≥ 5 high‐intensity sessions	160 (30.59%)	296 (25.58%)	467 355 (24.85%)	175 (33.59%)

Abbreviation: GAD, general anxiety disorder, OCD, obsessive‐compulsive disorder, PTSD, post‐traumatic stress disorder; SD, standard deviation. Individuals data items corresponding to less than 5 individuals were suppressed and replaced with a * for data confidentiality purposes.

### 3.2 Study aims

#### 3.2.1 Aim 1: Outcomes for people living with atypical dementia

Clinical measures and outcomes are presented in Table 2. People with atypical dementia experienced a moderate reduction in their symptoms of depression (Cohen *d* = −0.92 [−1.05 to −0.79]) and anxiety (*d* = −0.85 [−0.98 to −0.73]) after a course of treatment. They achieved a reliable improvement rate of 62.33%.

**TABLE 2:** Clinical measures and outcomes.

	Atypical dementia diagnosis	Typical dementia diagnosis	No dementia diagnosis
	*N* = 523	*N* = 1157	*N* = 1 880 357
	Mean (SD) range	Mean (SD) range	Mean (SD) Range
PHQ‐9—before treatment	16.02 (5.45) 0–27	15.85 (5.66) 0–27	15.85 (5.53) 0–27
PHQ‐9—after treatment	10.44 (6.64) 0–27	10.52 (6.89) 0–27	9.54 (6.85) 0–27
PHQ9—change	−5.57 (6.53) −26 to 14	−5.33 (6.56) −27 to 18	−6.31 (6.56) −27 to 27
GAD7—before treatment	13.17 (4.90) 0–21	13.19 (4.96) 0–21	14.35 (4.36) 0–21
GAD7 – after treatment	8.59 (5.81) 0–21	8.85 (5.91) 0–21	8.58 (5.94) 0–21
GAD7‐change	−4.58 (5.76) −21 to 11	−4.34 (5.67) −21 to 16	−5.76 (5.89) −21 to 21
	***n* (%)**	***n* (%)**	***n* (%)**
Reliable improvement (%)	326 (62.33%)	687 (59.38%)	1,302,747 (69.28%)
Recovery (%)	244 (46.65%)	530 (45.81%)	929,305 (49.42%)
Reliable recovery (%)	217 (41.49%)	466 (40.28%)	886,452 (47.14%)
Reliable deterioration (%)	43 (8.22%)	94 (8.12%)	109,294 (5.81%)
Reasons for ending treatment			
Completed	300 (57.36%)	601 (51.94%)	909,216 (48.35%)
Dropout	110 (21.03%)	252 (21.78%)	409,957 (21.80%)
Service not suitable^a^	13 (2.49%)	34 (2.94%)	17,301 (0.92%)
Declined	16 (3.06%)	28 (2.42%)	51,010 (2.71%)
Referred on^a^	26 (4.97%)	76 (6.57%)	63,137 (3.36%)
Missing	58 (11.09%)	166 (14.35%)	429,736 (22.85%)

Abbreviations: GAD, General Anxiety Disorder; PHQ, Patient Health Questionnaire; SD, standard deviation.

^a^ Categories “Service not suitable” and “Referred on” were pooled in Aim 4 analyses using reason for discharge as an outcome because of small sample sizes.

#### 3.2.2 Aim 2: Pretreatment factors associated with reliable improvement for people living with atypical dementia

In the multivariable logistic regression, age > 65 (odds ratio [OR] = 1.65 [1.02; 2.68]), experiencing moderate or severe (as opposed to mild) depression (OR = 1.39 [1.11; 1.74]) or anxiety (OR = 1.52 [1.19; 1.95]) symptoms and receiving five or more high‐intensity (OR = 1.97 [1.54 to 2.52]) or low‐intensity sessions (OR = 1.81 [1.33 to 2.46]) were associated with greater odds of reliable improvement. In contrast, living in the most deprived areas (lowest IMD quintile; OR = 0.65 [0.51 to 0.82]) and waiting > 6 weeks between referral and assessment (OR = 0.65 [0.48 to 0.87]) were associated with lower odds of reliable improvement (see Appendix S7.3 in supporting information for the full model).

#### 3.2.3 Aim 3: Comparisons of outcomes for people with typical dementia or without dementia

Overall, there was no evidence of a difference in the odds of reliable improvement between people with atypical dementia and those with typical dementia (OR = 1.07, 95% confidence interval [CI]: 0.85 to 1.34; Table 3).

**TABLE 3:** Reliable improvement for people living with atypical dementia, compared to people with a typical dementia diagnosis or no dementia diagnosis.

	Atypical dementia vs. typical dementia		Atypical dementia vs. no dementia	
	*N* = 1680		*N* = 1,880,880	
	**OR (95% CI)**	***p* value**	**OR (95% CI)**	***p* value**
Model 1: unadjusted	1.13 (0.92; 1.40)	0.2518	0.73 (0.61; 0.88)	0.0006
Model 2: fully adjusted	1.08 (0.86; 1.34)	0.5328	0.68 (0.56; 0.81)	<0.0001
Model 3: matched fully adjusted	N/A	N/A	*N* = 1042 0.70 (0.53; 0.91)	0.0150

*Note*: Model 2 included age (categorical), sex, Index of Multiple Deprivation, ethnicity, taking psychotropic medications, self‐reporting long‐term health conditions, baseline depression and anxiety scores, number of low‐intensity sessions, number of high‐intensity sessions, waiting times from referral to assessment and assessment to treatment, appointment year as covariates. Model 3 was run using the matched cohort including the same covariates as Model 2, as well as a measure of NHS Integrated Care Board as a fixed categorical covariate.

Abbreviations: CI, confidence interval; N/A, not applicable, OR, odds ratio.

A total of 521 out of the 523 individuals in the cohort of people with atypical dementia were matched to an individual without any diagnosis of dementia. The matched cohort of people living without dementia had similar characteristics to the cohort of people living with atypical dementia (Table 1).

People with atypical dementia were less likely to experience a reliable improvement compared to those without dementia (both before and after propensity score matching; Model 3: OR = 0.70, 95% CI: 0.53 to 0.91).

#### 3.2.4 Aim 4: Comparisons of reasons for discharge between people with typical dementia or without dementia

Reasons for discharge were similar between people with atypical and typical dementias (Table 2). Before matching, people with atypical dementia were more likely to be referred to other services than people living without dementia (OR = 1.51, 95% CI: 1.07 to 2.13), but this difference was no longer observed after matching (Table 4).

**TABLE 4:** Reason for discharge—multinomial regression.

	Atypical dementia vs. typical dementia		Atypical dementia vs. no dementia	
	***N* = 1680**		***N* = 1,880,880**	
	RRR (95% CI)	*p* value	RRR (95% CI)	*p* value
**Adjusted as in Model 2**				
Completed (ref)	Ref		Ref	
Dropout or declined	0.92 (0.69; 1.21)	0.5360	1.05 (0.84; 1.32)	0.6623
Referred on or service not suitable	0.77 (0.50; 1.18)	0.2295	1.51 (1.07; 2.13)	0.0204
Missing	0.78 (0.50; 1.23)	0.2905	1.02 (0.67; 1.57)	0.9172
**Matched cohort, Model 3**	N/A		*N* = 1042	
Completed (ref)			Ref	
Dropout or declined			1.07 (0.77; 1.49)	0.6939
Referred on or service not suitable			1.02 (0.60; 1.72)	0.9501
Missing			0.78 (0.45; 1.35)	0.3802

*Note*: Model 2 included age (categorical), sex, Index of Multiple Deprivation, ethnicity, taking psychotropic medications, self‐reporting long‐term health conditions, baseline depression and anxiety scores, number of low‐intensity sessions, number of high‐intensity sessions, waiting times from referral to assessment and assessment to treatment, appointment year as covariates. Model 3 was run using the matched cohort including the same covariates as Model 2, as well as a measure of NHS Integrated Care Board as a fixed categorical covariate.

Abbreviations: CI, confidence interval; N/A, not applicable, RRR, relative risk ratio.

### 3.3 Sensitivity analyses

Sensitivity analyses including an additional 630 people with ICD‐10 code G31.9 yielded similar results (see Appendix S7).

### 3.4 Supplementary analyses

Analyses were also conducted using reliable recovery and reliable deterioration as outcomes.

For Aim 2, in the logistic regression, waiting > 6 weeks between assessment and treatment (OR = 0.38, 95% CI: 0.21 to 0.71) and self‐reporting a long‐term health condition (OR = 0.61, 95% CI: 0.39 to 0.96) were associated with lower odds of reliable recovery, while receiving five or more low‐intensity (OR = 1.72, 95% CI: 1.04 to 2.86) or high‐intensity (OR = 1.96, 95% CI: 1.31 to 2.94) treatment sessions were associated with higher odds of reliable recovery. Experiencing moderate or severe depression symptoms was associated with higher odds of reliable deterioration (OR = 0.34, 95% CI: 0.17 to 0.68; see Appendix S8 in supporting information). Due to the lower rates of recovery and deterioration observed, compared to reliable improvement, the predictor analyses had less statistical power than the reliable improvement analyses, and this may have led to the identification of fewer meaningful predictors.

People with atypical dementia were as likely to meet the criteria for reliable recovery (OR = 1.05, 95% CI: 0.84 to 1.32, *p* = 0.6573) and reliable deterioration (OR = 1.04, 95% CI: 0.71 to 1.53, *p* = 0.8570) as people with typical dementia. In the matched analyses, people with atypical dementia were considerably less likely to meet reliable recovery (OR = 0.57, 95% CI: 0.44 to 0.75), and as likely to meet criteria for reliable deterioration (OR = 1.08, 95% CI: 0.67 to 1.75, *p* = 0.7572) as people without dementia.

## 4 DISCUSSION

Using a cohort of national coverage in England, we found that people diagnosed with atypical dementias who accessed primary care psychological therapy services when experiencing depression or anxiety experienced a reduction in their symptoms, with moderate effect sizes. As we have reported previously,^15^ people living with dementia have less favorable outcomes than those without dementia, but they do nonetheless benefit. This study suggests that despite the different symptom profiles of atypical dementias, they were no less likely to experience an improvement in their depression and anxiety symptoms than people with typical dementias, with similar reasons for ending treatment. There has been relatively little research exploring how therapy can be optimized for people with dementia, perhaps reflecting a long‐held nihilism about whether this group was able to benefit. Research is required to understand how outcomes could be improved for people with typical and atypical dementias, whose different symptom profiles may require different adaptations.

As in earlier studies, we found that older age^17^, ^24^ and lower level social deprivation^31^ may be associated with better therapy outcomes in people living with, or without, dementia. Perhaps, in line with previous findings regarding treatment access, people from less deprived backgrounds may experience better outcomes because they experience less stress due to economic hardship.^32^ People from less deprived backgrounds may also access dementia care earlier,^33^ and access therapy when symptoms are less severe and have resulted in fewer social and interpersonal sequelae, such as disengagement from supportive relationships. One of the factors strongly associated with good therapy response was waiting < 6 weeks between referral and assessment. In other words, in this study, prompt treatment appeared to matter more than dementia subtype. People with atypical dementias have reported experiencing long and complicated pathways to the diagnosis of their dementia, with many reporting multiple or inappropriate referrals before accessing diagnosis.^34^ In light of these experiences, it is possible that this population may be specifically vulnerable to longer waiting times when being referred to a new service when experiencing mental health difficulties. This could in part be due to the potential deterioration of cognitive symptoms of dementia associated with longer waiting times, which may have a potential adverse impact on therapy outcomes.

A higher number of therapy sessions has previously been linked with better outcomes,^17^ although as in all observational research, causality is unclear. It is possible that receiving more treatment sessions leads to better therapy outcomes; alternatively, people may receive more treatment sessions and remain engaged in therapy because their symptoms improve.

This study has several limitations, due to constraints inherent to the use of electronic health‐care records for this study. First, although the PHQ‐9 and the GAD‐7 have been used in populations of people living with dementia, they have not been fully validated for these populations, meaning that our study findings may not capture symptoms associated with dementia‐specific experiences of depression or anxiety. For example, FTD is associated with changes in personality and emotional processing,^35^ which may affect how people experience depression and anxiety, how they report symptoms, and how they respond to psychological therapy. Second, diagnostic coding of specific subtypes of atypical dementias may not be straightforward using the ICD‐10 code classification system,^36^ which leads to a lack of equivalence between neuropsychology‐specific diagnostic criteria and diagnostic coding using this classification system. This means that our cohort may not be representative of the population of people living with an atypical dementia who access primary care psychological therapy services, and that some people who may have undiagnosed dementia may have been included in the group without dementia. Moreover, the lack of granularity in the ICD‐10 codes used in this study means that analyses by subtype of dementia would not be feasible; that is, this study could not evaluate outcomes in people with specific subtypes of atypical dementia such as PCA, primary progressive aphasia, or DLB. This is a limitation that may partially be addressed in future research with the introduction of a PCA diagnostic code in the 11th version of the ICD classification system.^37^ Similarly, although we suspect that people receiving a course of therapy were in the early stages and experiencing mild to moderate symptoms of dementia, such information was not available. This means that we were unable to assess the impact of specific dementia symptoms such as differences in speech or visual processing, or how dementia severity may have affected therapy outcomes. It is also unknown whether and to what extent adaptations may have been provided to those people with atypical dementias who received a course of therapy. Finally, people living with dementia represented 0.09% of the total study cohort. Although prevalence data by subtype is not available, this figure is well below the expected prevalence of dementia in the general population. It suggests that many people living with dementia (whether typical or atypical) may not access therapy or may be misclassified in the cohort due to issues with ICD‐10 code reporting outlined above. This calls for a better understanding of how people living with atypical dementia experience access to psychological services, and the care pathways they go through when seeking support for their mental health.

Overall, these findings suggest that people living with atypical dementia may benefit from being referred to primary care psychological therapy services when experiencing depression or anxiety.

Neuropsychiatric symptoms have been associated with disease progression in non–memory‐led dementia such as PCA^38^ and FTD.^39^, ^40^ This means that improving access to psychological therapy services may play an instrumental role in these patients’ care and well‐being.

Evidence indicates that people with typical and atypical dementia benefit from therapy. Findings suggest a need to provide assessments and start care sooner for these patients, and that an expansion of services may offer positive benefits to people with both typical and atypical dementia, a group for whom there are few evidence‐based treatments, especially as assessment delay is associated with worse outcomes. As limited research has explored how to optimize therapies for this group, greater benefits may follow further work on the specific adaptations and their implementation. Such research should investigate how specific symptoms or disease severity may affect therapy outcomes as well as access to services.

## AUTHOR CONTRIBUTIONS

Celine El Baou, Emilie Brotherhood, Amber John, Joshua Stott, Rob Saunders, Marcus Richards and Claudia Cooper were involved in the conceptualization and design of the study. Celine El Baou, Emilie Brotherhood, Amber John, Joshua Stott, Rob Saunders, and Joshua E. J. Buckman contributed to the methodology. Emilie Brotherhood, Sebastian Crutch, and Joshua Stott were involved in the atypical dementia ascertainment consensus meeting. Amber John, Rob Saunders, and Celine El Baou were involved in data extraction and acquisition. Celine El Baou carried out the analyses. Celine El Baou, Amber John, and Rob Saunders assessed and verified the underlying data reported in the manuscript. Natalie L. Marchant and Val Mansfield were PPI representatives. Celine El Baou wrote the first draft of the manuscript, and all authors contributed to the writing and review of subsequent versions of the manuscript and approved the final version. All authors had full access to all data in the study and accept the responsibility to submit for publication.

## CONFLICTS OF INTERESTS STATEMENT

G.B., J.S., and A.J. are supported by the Alzheimer's Society (AS‐PG‐18‐013). J.E.J.B. and R.S. are supported by the Royal College of Psychiatrists. R.S. held an unrelated honorary position with NHS England, and their time was compensated through financial support to the employing institution. R.S. is on the editorial board of the *British Journal of Psychiatry*. J.S. has been a consultant to NHS Wales Shared Services Partnership and is involved in unrelated research projects funded by the National Institute for Health and Care Research (NIHR) Public Health Research, Dunhill Medical Trust, and Economic and Social Research Council/NIHR. All other authors have no interests to declare. Author disclosures are available in the supporting information.

## CONSENT STATEMENT

Records were pseudonymized and linked using a key provided by NHS Digital; as such, informed consent was not necessary for this study.

## Supporting information



Supporting Information

Supporting Information

## Data Availability

All data used for this study are available upon successful application to NHS Digital via the Data Access Request Service (DARS): https://digital.nhs.uk/services/data‐access‐request‐service‐dars. Data fields can be accessed via the NHS Digital data dictionary: https://www.datadictionary.nhs.uk/.
