# Supplementary material for: Effectiveness of psychological therapies for depression and anxiety in atypical dementia
Source: Alzheimers Dement. 2024 Oct 23;20(12):8844–54. doi: 10.1002/alz.14332 (PMC11667512; doi:10.1002/alz.14332)
Supplement: Supplementary file 1 — Supporting Information [file ALZ-20-8844-s002.docx]

**APPENDIX**

[Appendix 1 MODIFY Study Dataset 2](#_Toc177722301)

[Appendix 2 NHS Talking Therapies for Anxiety and Depression Services 3](#_Toc177722302)

[Appendix 3 The RECORD statement – checklist of items, applied to the present study 5](#_Toc177722303)

[Appendix 4 Typical and atypical dementia diagnosis ascertainment 13](#_Toc177722304)

[Appendix 5 Covariates used in analyses 15](#_Toc177722305)

[Appendix 6 Matching Procedure 17](#_Toc177722306)

[Appendix 7 Sensitivity analyses – including G31.9 in the atypical dementia group 18](#_Toc177722307)

[Appendix 8 Supplementary analyses – Factors associated with reliable recovery and reliable deterioration for people with atypical dementia, multivariable logistic regression 23](#_Toc177722308)

# MODIFY Study Dataset

| **NHS TTad (formerly IAPT) dataset [1]** | Routinely collected data for every patient seen in IAPT services across all 211 clinical commissioning group areas in England between 2012 (the date of database inception) to 2019 (the latest data point available at time of analysis. Includes demographic (e.g., gender, age, ethnicity), therapy (e.g., referral and assessment dates, treatment information at each appointment) and outcome (improvement, recovery, deterioration) information for individual patients. |
| --- | --- |
| **Hospital Episode Statistics dataset [2]** | Admitted Patient Car and Outpatient datasets from all National Health Service (NHS) hospitals across England. Includes demographic (e.g., age, ethnicity, gender), geographical (e.g., residential area, area treatment received), administrative (e.g., dates of admission and discharge), and clinical (e.g., diagnoses, treatments, operations) information for individual patients. |
| **Mental Health Services dataset [3]** | Previously known as the Mental Health Minimum Dataset (MHMDS) and the Mental Health and Learning Disability Dataset (MHLDDS). Includes data from secondary care services (e.g., provided in hospitals, outpatient clinics, in the community) for mental illness, learning disability, autism, and other neurodevelopmental conditions. |
| **HES-ONS Mortality dataset [4]** | Linked information from HES and Office of National Statistics (ONS) mortality data. Includes cause, date, and place of death (both in and out of hospital). |

# NHS Talking Therapies for Anxiety and Depression Services

After an initial assessment, and prior to treatment start, the clinician and the person receiving treatment agree on which condition therapy will focus on. Many people will meet diagnostic criteria for several mental health conditions, and our dataset captures information on the condition being treated. People with less complex problems are commonly offered short-term (<=8 sessions) “low intensity” guided self-help therapies, which can be delivered face to face or remotely, in one-to-one or group settings. Where people do not respond to low intensity therapy or their problems are more complex at referral, individuals are commonly offered high intensity therapies (>=16-20 sessions), which are formulation driven one-to-one cognitive behavioural or other types of evidence-based therapies. Interventions are standardized and delivered by trained practitioners and psychotherapists following evidence-based protocols[5].

|  | **Condition** | **Psychological therapies** | **Source** |
| --- | --- | --- | --- |
| Step 2: Low-intensity interventions | Depression | Individual guided self-help based on cognitive behavioural therapy (CBT), computerised CBT, behavioural activation, structured group physical activity programme | NICE guidelines NG222, CG91, CG123 |
|  | Generalised anxiety disorder | Self-help, or guided self-help based on CBT, psycho-educational groups, computerised CBT | NICE guidelines CG113, CG123 |
|  | Panic disorder | Self-help, or guided self-help based on CBT, psycho-educational groups, computerised CBT | NICE guidelines CG31, CG123 |
|  | Obsessive-compulsive disorder | Guided self-help based on CBT | NICE guidelines CG31, CG123 |
| Step 3: High intensity interventions | Depression  For individuals with mild to moderate severity who have not responded to initial low-intensity interventions | CBT (individual or group) or interpersonal therapy (IPT)  Behavioural Activation  Couple therapy  Counselling for depression  Brief psychodynamic therapy  Note: Psychological interventions can be provided in combination with antidepressant medication | NICE guidelines: NG222, CG91, CG123 |
|  | Depression  Moderate to severe | CBT (individual) or IPT, each with medication | NICE guidelines: NG222, CG91, CG123 |

Source: National Collaborating Centre for Mental Health. The Improving Access to Psychological Therapies Manual 2021.

# The RECORD statement – checklist of items, applied to the present study

|  | **Item No.** | **STROBE items** | **Location in manuscript where items are reported** | **RECORD items** | **Location in manuscript where items are reported** |  |
| --- | --- | --- | --- | --- | --- | --- |
| **Title and abstract** | | | | | | |
|  | 1 | (a) Indicate the study’s design with a commonly used term in the title or the abstract (b) Provide in the abstract an informative and balanced summary of what was done and what was found | Methods – Data sources and procedures | RECORD 1.1: The type of data used should be specified in the title or abstract. When possible, the name of the databases used should be included.  RECORD 1.2: If applicable, the geographic region and timeframe within which the study took place should be reported in the title or abstract.  RECORD 1.3: If linkage between databases was conducted for the study, this should be clearly stated in the title or abstract. | Methods – Data sources and procedures  Title, Methods – Data sources and procedures  Methods – Data sources and procedures |  |
| **Introduction** | | | | | | |
| Background rationale | 2 | Explain the scientific background and rationale for the investigation being reported | Introduction |  | Introduction |  |
| Objectives | 3 | State specific objectives, including any prespecified hypotheses | Research aims 1 to 4 |  | Research aims 1 to 4 |  |
| **Methods** | | | | | | |
| Study Design | 4 | Present key elements of study design early in the paper | Methods – Data sources and procedures |  | Methods – Data sources and procedures |  |
| Setting | 5 | Describe the setting, locations, and relevant dates, including periods of recruitment, exposure, follow-up, and data collection | Methods – Data sources and procedures, appendix A, |  |  |  |
| Participants | 6 | *(a) Case-control study* - Give the eligibility criteria, and the sources and methods of case ascertainment and control selection. Give the rationale for the choice of cases and controls  *(b) Case-control study* - For matched studies, give matching criteria and the number of controls per case | Methods / Study Population, Dementia Ascertainment, Appendix C | RECORD 6.1: The methods of study population selection (such as codes or algorithms used to identify subjects) should be listed in detail. If this is not possible, an explanation should be provided.  RECORD 6.2: Any validation studies of the codes or algorithms used to select the population should be referenced. If validation was conducted for this study and not published elsewhere, detailed methods and results should be provided.  RECORD 6.3: If the study involved linkage of databases, consider use of a flow diagram or other graphical display to demonstrate the data linkage process, including the number of individuals with linked data at each stage. | Methods / Study Population, Dementia Ascertainment, Appendix C  Methods / Study Population, Dementia Ascertainment, Appendix C  Methods / Study Population |  |
| Variables | 7 | Clearly define all outcomes, exposures, predictors, potential confounders, and effect modifiers. Give diagnostic criteria, if applicable. | Method / Dementia Ascertainment, Outcomes, Covariates | RECORD 7.1: A complete list of codes and algorithms used to classify exposures, outcomes, confounders, and effect modifiers should be provided. If these cannot be reported, an explanation should be provided. | Method/ Dementia Ascertainment, Outcomes, Covariates, Appendix C, Appendix D |  |
| Data sources/ measurement | 8 | For each variable of interest, give sources of data and details of methods of assessment (measurement).  Describe comparability of assessment methods if there is more than one group | Appendix D Methods / Outcome measures / Covariates / Sensitivity |  |  |  |
| Bias | 9 | Describe any efforts to address potential sources of bias | Methods / Statistical Analysis, Appendix E |  |  |  |
| Study size | 10 | Explain how the study size was arrived at | Method/Data sources and procedures |  |  |  |
| Quantitative variables | 11 | Explain how quantitative variables were handled in the analyses. If applicable, describe which groupings were chosen, and why | Method / Dementia Ascertainment / Statistical Analyses / Appendix D, Appendix E |  |  |  |
| Statistical methods | 12 | (a) Describe all statistical methods, including those used to control for confounding  (b) Describe any methods used to examine subgroups and interactions  (c) Explain how missing data were addressed  (d) *Case-control study* - If applicable, explain how matching of cases and controls was addressed  (e) Describe any sensitivity analyses | (a) Methods / Statistical Analysis, Appendix  (b) Methods / Statistical Analysis  (c) Methods / Statistical Analysis  (d) Methods / Statistical Analysis Appendix E  (e)Methods / Statistical Analysis , Appendix F | | |  |
| Data access and cleaning methods |  | .. |  | RECORD 12.1: Authors should describe the extent to which the investigators had access to the database population used to create the study population.  RECORD 12.2: Authors should provide information on the data cleaning methods used in the study. | Authors contribution  Methods / Data sources and procedures |  |
| Linkage |  | .. |  | RECORD 12.3: State whether the study included person-level, institutional-level, or other data linkage across two or more databases. The methods of linkage and methods of linkage quality evaluation should be provided. | Methods / Data sources and procedures, Appendix B, Appendix C, Appendix D |  |
| **Results** | | | | | | |
| Participants | 13 | (a) Report the numbers of individuals at each stage of the study (*e.g.*, numbers potentially eligible, examined for eligibility, confirmed eligible, included in the study, completing follow-up, and analysed)  (b) Give reasons for non-participation at each stage.  (c) Consider use of a flow diagram | Results/Demographic and sample characteristics | RECORD 13.1: Describe in detail the selection of the persons included in the study (*i.e.,* study population selection) including filtering based on data quality, data availability and linkage. The selection of included persons can be described in the text and/or by means of the study flow diagram. | Results/Demographic and sample characteristics |  |
| Descriptive data | 14 | (a) Give characteristics of study participants (*e.g.*, demographic, clinical, social) and information on exposures and potential confounders  (b) Indicate the number of participants with missing data for each variable of interest | (a, b) Table 1 |  |  |  |
| Outcome data | 15 | *Case-control study* - Report numbers in each exposure category, or summary measures of exposure  *Cross-sectional study* - Report numbers of outcome events or summary measures | Table 1 |  |  |  |
| Main results | 16 | (a) Give unadjusted estimates and, if applicable, confounder-adjusted estimates and their precision (e.g., 95% confidence interval). Make clear which confounders were adjusted for and why they were included  (b) Report category boundaries when continuous variables were categorized  (c) If relevant, consider translating estimates of relative risk into absolute risk for a meaningful time period | Table 2, Table 3  Table 1  Not applicable |  |  |  |
| Other analyses | 17 | Report other analyses done—e.g., analyses of subgroups and interactions, and sensitivity analyses | Results, Appendix F |  |  |  |
| **Discussion** | | | | | | |
| Key results | 18 | Summarise key results with reference to study objectives | Discussion 1^st^ Paragraph |  |  |  |
| Limitations | 19 | Discuss limitations of the study, taking into account sources of potential bias or imprecision. Discuss both direction and magnitude of any potential bias | Discussion / Limitations | RECORD 19.1: Discuss the implications of using data that were not created or collected to answer the specific research question(s). Include discussion of misclassification bias, unmeasured confounding, missing data, and changing eligibility over time, as they pertain to the study being reported. | Discussion / Limitations |  |
| Interpretation | 20 | Give a cautious overall interpretation of results considering objectives, limitations, multiplicity of analyses, results from similar studies, and other relevant evidence | Discussion / 1^s^ section, Limitations |  |  |  |
| Generalisability | 21 | Discuss the generalisability (external validity) of the study results | Discussion, 1^st^ section |  |  |  |
| **Other Information** | | | | | | |
| Funding | 22 | Give the source of funding and the role of the funders for the present study and, if applicable, for the original study on which the present article is based | Funding |  |  |  |
| Accessibility of protocol, raw data, and programming code |  | .. |  | RECORD 22.1: Authors should provide information on how to access any supplemental information such as the study protocol, raw data, or programming code. | N/A |  |

*Reference: Benchimol EI, Smeeth L, Guttmann A, Harron K, Moher D, Petersen I, Sørensen HT, von Elm E, Langan SM, the RECORD Working Committee. The REporting of studies Conducted using Observational Routinely-collected health Data (RECORD) Statement. *PLoS Medicine* 2015; in press.

*Checklist is protected under Creative Commons Attribution ([CC BY](http://creativecommons.org/licenses/by/4.0/)) license.

# Typical and atypical dementia diagnosis ascertainment

**Atypical dementia, code list** **after consensus meeting.**

| **ICD-10 Code** | **ICD-10 description** |
| --- | --- |
| F00.2 | Dementia in Alzheimer disease, atypical or mixed type |
| F02.0 | Dementia in Picks disease |
| F02.2 | Dementia in Huntington’s disease |
| F02.3 | Dementia in Parkinson's disease |
| F02.4 | Dementia in HIV disease |
| G10 | Huntington's disease |
| G23.1 | Progressive supranuclear palsy |
| G30.8 | Other Alzheimer disease |
| G31.0 | Circumscribed brain atrophy |
| G31.1 | Senile degeneration of brain, not elsewhere classified  Excluded:  Alzheimer disease (G30.-)  senility NOS (R54) |
| G31.8 | Other specified degenerative diseases of nervous system  Grey-matter degeneration [Alpers]  Lewy body(ies)(dementia)(disease) (F02.8*)  Subacute necrotizing encephalopathy [Leigh] |

Additionally included in sensitivity analyses: G31.9 Degenerative disease of nervous system, unspecified.

**Typical dementia, code list**

The code list will be based on codes used in the Hospital Episode Statistics validation study^6^

| **ICD-10 codes** | **ICD-10 code description** |
| --- | --- |
| E512 | Wernicke encephalopathy |
| F00 | Dementia in Alzheimer disease |
| F01 | Vascular dementia |
| F02 | Dementia in other diseases classified elsewhere |
| F03 | Unspecified dementia |
| F10.6 | Mental and behavioural disorders due to use of alcohol (Amnesic syndrome) |
| F10.7 | Mental and behavioural disorders due to use of alcohol (Residual and late-onset psychotic disorder) |
| G30 | Alzheimer disease |
| G31.0 | Circumscribed brain atrophy |

Were excluded from this list those codes that relate to atypical dementias as defined above:

| **ICD-10 codes** | **ICD-10 code description** |
| --- | --- |
| F00.2 | Dementia in Alzheimer disease, atypical or mixed type |
| F02.0 | Dementia in Picks disease |
| F02.2 | Dementia in Huntington’s disease |
| F02.3 | Dementia in Parkinson's disease |
| F02.4 | Dementia in HIV disease |
| G30.8 | Other Alzheimer disease |
| G31.0 | Circumscribed brain atrophy |

# Covariates used in analyses

A range of covariates known to be associated with therapy outcomes[6-10] were included in analyses, with the constraint of data availability. Variables were fitted as a categorical variable in the model when a non-linear association with therapy outcomes was found. Categories were defined based on previous research or NHS clinical standards (e.g age < 65).

| **Variables** | **Identification in databases** | Data source |
| --- | --- | --- |
|  |  |  |
|  |  |  |
|  |  |  |
| **Socio-demographic pre-treatment factors** | | |
| Demographic covariates | Self-reported measures collected at the point of referral: gender, age, index of multiple deprivation (IMD) quintile (a lowest IMD indicates a higher deprivation area), ethnicity (based on UK census codes ‘White’, ‘Mixed’, ‘Asian’, ‘Black’, ‘Chinese’ and ‘other’), and employment status ( employed, unemployed and seeking, unemployed and not seeking work, unemployed, long-term sickness or disability) were available in the dataset. | NHS TTad |
| Long-term health conditions | All patients are asked whether they have any long-term physical health condition (LTC) at referral. The type of condition was not available in the dataset. Presence of a long-term condition may be associated with an adaptation in the therapy provided. | NHS TTad |
| Psychotropic Medication taken at start of treatment | Clinicians in the services routinely record whether their patients were prescribed psychotropic medication(s) before treatment. | NHS TTad |
| **Service and therapy factors** | |  |
| NHS TTad treatment factors | The number of treatment sessions received, Reason for treatment discontinuation (Completed, Dropout, Not suitable, Declined, referred to another service), Year of referral, Number if high intensity treatment session, number of low intensity treatment sessions, frequency of sessions / week | NHS TTad |

Abbreviations: HES=Hospital Episode Statistics, NHS TTad = Improving Access to Psychological Therapies, ICD = International Classification of Disease, MHSDS=Mental Health Services Dataset, N/A = Not applicable, PS = Propensity Score

A measure of NHS Integrated Care Board (ICB) was also included in the propensity score matching algorithm:

| **ICB Code** | **Integrated Care Board Name** |
| --- | --- |
| QKK | NHS SOUTH EAST LONDON INTEGRATED CARE BOARD |
| QMF | NHS NORTH EAST LONDON INTEGRATED CARE BOARD |
| QMJ | NHS NORTH CENTRAL LONDON INTEGRATED CARE BOARD |
| QRV | NHS NORTH WEST LONDON INTEGRATED CARE BOARD |
| QWE | NHS SOUTH WEST LONDON INTEGRATED CARE BOARD |
| QJK | NHS DEVON INTEGRATED CARE BOARD |
| QOX | NHS BATH AND NORTH EAST SOMERSET, SWINDON AND WILTSHIRE INTEGRATED CARE BOARD |
| QR1 | NHS GLOUCESTERSHIRE INTEGRATED CARE BOARD |
| QSL | NHS SOMERSET INTEGRATED CARE BOARD |
| QT6 | NHS CORNWALL AND THE ISLES OF SCILLY INTEGRATED CARE BOARD |
| QUY | NHS BRISTOL, NORTH SOMERSET AND SOUTH GLOUCESTERSHIRE INTEGRATED CARE BOARD |
| QVV | NHS DORSET INTEGRATED CARE BOARD |
| QKS | NHS KENT AND MEDWAY INTEGRATED CARE BOARD |
| QNQ | NHS FRIMLEY INTEGRATED CARE BOARD |
| QNX | NHS SUSSEX INTEGRATED CARE BOARD |
| QRL | NHS HAMPSHIRE AND ISLE OF WIGHT INTEGRATED CARE BOARD |
| QU9 | NHS BUCKINGHAMSHIRE, OXFORDSHIRE AND BERKSHIRE WEST INTEGRATED CARE BOARD |
| QXU | NHS SURREY HEARTLANDS INTEGRATED CARE BOARD |
| QGH | NHS HEREFORDSHIRE AND WORCESTERSHIRE INTEGRATED CARE BOARD |
| QHL | NHS BIRMINGHAM AND SOLIHULL INTEGRATED CARE BOARD |
| QJ2 | NHS DERBY AND DERBYSHIRE INTEGRATED CARE BOARD |
| QJM | NHS LINCOLNSHIRE INTEGRATED CARE BOARD |
| QK1 | NHS LEICESTER, LEICESTERSHIRE AND RUTLAND INTEGRATED CARE BOARD |
| QNC | NHS STAFFORDSHIRE AND STOKE-ON-TRENT INTEGRATED CARE BOARD |
| QOC | NHS SHROPSHIRE, TELFORD AND WREKIN INTEGRATED CARE BOARD |
| QPM | NHS NORTHAMPTONSHIRE INTEGRATED CARE BOARD |
| QT1 | NHS NOTTINGHAM AND NOTTINGHAMSHIRE INTEGRATED CARE BOARD |
| QUA | NHS BLACK COUNTRY INTEGRATED CARE BOARD |
| QWU | NHS COVENTRY AND WARWICKSHIRE INTEGRATED CARE BOARD |
| QH8 | NHS MID AND SOUTH ESSEX INTEGRATED CARE BOARD |
| QH8 | NHS MID AND SOUTH ESSEX INTEGRATED CARE BOARD |
| QHG | NHS BEDFORDSHIRE, LUTON AND MILTON KEYNES INTEGRATED CARE BOARD |
| QJG | NHS SUFFOLK AND NORTH EAST ESSEX INTEGRATED CARE BOARD |
| QM7 | NHS HERTFORDSHIRE AND WEST ESSEX INTEGRATED CARE BOARD |
| QMM | NHS NORFOLK AND WAVENEY INTEGRATED CARE BOARD |
| QUE | NHS CAMBRIDGESHIRE AND PETERBOROUGH INTEGRATED CARE BOARD |
| QE1 | NHS LANCASHIRE AND SOUTH CUMBRIA INTEGRATED CARE BOARD |
| QOP | NHS GREATER MANCHESTER INTEGRATED CARE BOARD |
| QYG | NHS CHESHIRE AND MERSEYSIDE INTEGRATED CARE BOARD |
| QF7 | NHS SOUTH YORKSHIRE INTEGRATED CARE BOARD |
| QHM | NHS NORTH EAST AND NORTH CUMBRIA INTEGRATED CARE BOARD |
| QOQ | NHS HUMBER AND NORTH YORKSHIRE INTEGRATED CARE BOARD |
| QWO | NHS WEST YORKSHIRE INTEGRATED CARE BOARD |

# Matching Procedure

To take potential regional clustering effect into account, the matching algorithm occurred in two steps. The propensity score was estimated using a logistic regression model, including atypical dementia (vs no dementia) as the outcome, and all available variables as covariates. To account for potential regional clustering effect, NHS Integrated Care Board was included as a fixed effect in both the propensity score estimation and adjusted for as a covariate in the outcome regression to reduce the risk of bias[11]. Exact matching was also used based on NHS Integrated Care Board.

The quality of the matching was assessed by comparing demographic characteristics of the atypical and no dementia group before and after matching. Where a control was identified as an appropriate match for more than one participant in the atypical sample, these were weighted and used in the analysis (maximum weight = 2), and a robust variance estimator was used in regressions.

# Sensitivity analyses – including G31.9 in the atypical dementia group

Results from main analyses are presented side-by-side for comparison.

**Table 7.1 Demographics, baseline characteristics and outcomes**

| **Demographic and baseline measures** |  | |
| --- | --- | --- |
|  | **Atypical Dementia**  **(broad def)** | **Atypical Dementia (from primary analyses)** |
|  | **N=1153** | **N=523** |
| Age at referral – Mean (SD) Range | 61.2 (16.2) 19-98 | 61.0 (17.05) 19-94 |
|  | **n (%)** | **n (%)** |
| **Age Category** |  |  |
| 65+ | 541 (46.92%) | 260 (49.71%) |
| **Ethnicity** | | |
| White | 946 (82.05%) | 430 (82.22%) |
| Mixed | 9 (0.78%) | 4 (0.76%0 |
| Asian | 56 (4.86%) | 11 (2.10%) |
| Black | 21 (1.82%) | 11 (2.10%) |
| Chinese | 0 (0.0%) | 5 (0.96%) |
| Other | 8 (0.69%) | 0 (0.0%) |
| Missing | 113 (9.80%) | 62 (11.85%) |
| **Gender** | | |
| Male | 518 (44.93%) | 223 (42.64%) |
| Female | 635 (55.07%) | 300 (57.36%) |
| Missing | 0 | 0 |
| **Index of multiple deprivation (quintile)** |  |  |
| 1 (Most deprived) | 292 (25.33%) | 114 (21.80%) |
| 2 | 218 (18.91%) | 105 (20.08%) |
| 3 | 243 (21.08%) | 117 (22.37%) |
| 4 | 197 (17.09%) | 97 (18.55%) |
| 5 (Least deprived) | 174 (15.09%) | 73 (13.96%) |
| Missing | 29 (2.52%) | 17 (3.25%) |
| **Taking psychotropic medication before therapy** |  |  |
| No | 382 (33.13%) | 173 (33.08%) |
| Yes | 666 (57.76%) | 295 (56.41%) |
| Missing | 105 (9.11%) | 55 (10.52%) |
| **Self-reported long-term health condition** |  |  |
| No | 313 (27.15%) | 130 (24.86%) |
| Yes | 596 (51.69%) | 290 (55.45%) |
| Missing | 244 (21.16%) | 103 (19.69%) |
| **Diagnosis category** |  |  |
| Depression | 451 (39.12%) | 219 (41.87%) |
| Mixed anxiety and depressive disorder | 148 (12.84%) | 73 (13.96%) |
| GAD | 138 (11.97%) | 60 (11.47%) |
| OCD | 9 (0.78%) | 2 (0.38%) |
| PTSD | 24 (2.08%) | 8 (1.53%) |
| Phobic anxiety and panic disorder | 44 (3.82%) | 21 (4.02%) |
| Unspecified anxiety | 3 (0.26%) | 0 (0.0%) |
| Missing | 336 (29.14%) | 140 (26.77%) |
| **Waiting times** |  |  |
| Time between ref. and assessment (weeks) | 3.09 (3.92) 0-28 | 3.04 (4.10) 0-28 |
| Time between ref. And assessment >6weeks |  | 67 (12.81%) |
| Time between assessment and treatment (weeks) | 9.68 (11-17) 0-36 | 10.05 (11.11) 0-36 |
| Time between ref and treatment >18 weeks |  | 121 (23.14%) |
| **Number of sessions** |  |  |
| 6 or more low intensity sessions | 185 (16.08%) | 83 (15.87%) |
| 6 or more high intensity sessions | 317 (27.49%) | 160 (30.59%) |
| **Reasons for ending treatment** |  |  |
| Completed | 648 (56.20%) | 300 (57.36%) |
| Dropout | 246 (21.34%) | 110 (21.03%) |
| Service not suitable | 29 (2.52%) | 13 (2.49%) |
| Declined | 26 (2.25%) | 16 (3.06%) |
| Referred on | 59 (5.12%) | 26 (4.97%) |
| Missing | 145 (12.58%) | 58 (11.09%) |

**Table 7.2: Aim 1 – Pre-post effect sizes and outcomes**

|  |  | |
| --- | --- | --- |
|  | **Atypical Dementia**  **(broad def)** | **Atypical Dementia (narrow def without G31.9)**  [Primary analysis in manuscript] |
|  | **N=1153** | **N=523** |
| PHQ-9 – Before treatment | 15.84 (5.53) 0-27 | 16.02 (5.45) 0-27 |
| PHQ-9- After treatment | 10.20 (6.94) 0-27 | 10.44 (6.64) 0-27 |
| PHQ9- Change | -5.64 (6.84) -26 to 14 | -5.57 (6.53) -26 to 14 |
| Cohen’s d | -0.90 (-0.99; -0.81) | -0.92 (-1.05; -0.79) |
| GAD7 – Before treatment | 13.26 (4.79) 0-21 | 13.17 (4.90) 0-21 |
| GAD7 – after treatment | 8.43 (5.86) 0-21 | 8.59 (5.81) 0-21 |
| GAD7-Change | -4.83 (5.93) -21 to 24 | -4.58 (5.76) -21 to 11 |
| Cohen’s d | -0.90 (0.99; -0.82) | -0.85 (-0.98; -0.73) |
| Reliable improvement (%) | 746 (64.70%) | 326 (62.33%) |
| Recovery (%) | 554 (48.05%) | 244 (46.65%) |
| Reliable Recovery (%) | 497 (43.10%) | 217 (41.49%) |
| Reliable Deterioration (%) | 98 (8.50%) | 43 (8.22%) |

**Table 7.3: Aim 2 – Factors associated with reliable improvement for people with atypical dementia, multivariable logistic regression**

|  | **Atypical Dementia**  **(broad def)** | **N=1153** | **Atypical Dementia (narrow def without G31.9)**  [Primary analysis in manuscript] |  |
| --- | --- | --- | --- | --- |
|  | OR (95% CI) | p-value | **N=523**  OR (95% CI) |  |
| **Age Category** |  |  |  |  |
| <65 | Ref |  | Ref |  |
| 65+ | **2.02 (1.44; 2.84)** | **<.0001** | **1.65 (1.02; 2.68)** | **0.0428** |
| **Ethnicity** |  |  |  |  |
| White | Ref |  | Ref |  |
| Asian | 3.06 (1.46; 6.43) | <.0031 | 0.69 (0.19; 2.46) | 0.5647 |
| Black | 1.31 (0.50; 3.47) | <0.5804 | 2.78 (0.66; 11.77) | 0.1638 |
| Missing/Other | 0.79 (0.52; 1.19) | <0.2671 | 0.98 (0.54; 1.76) | 0.9369 |
| **Gender** |  |  |  |  |
| Male | Ref |  | Ref |  |
| Female | 1.16 (0.89; 1.50) | 0.2467 | 1.03 (0.70; 1.53) | 0.8651 |
| **IMD quintile** |  |  |  |  |
| 2-5 (Least deprived) | Ref |  | Ref |  |
| 1 (Most deprived) | **0.59 (0.44; 0.80)** | **0.0007** | **0.51 (0.32; 0.82)** | **0.0052** |
| Missing | **0.53 (0.24; 1.15)** | **0.1081** | **0.84 (0.29; 2.44)** | **0.7421** |
| **Taking Psychotropic medication** |  |  |  |  |
| No | Ref |  | Ref |  |
| Yes | 0.99 (0.75; 1.32) | 0.2467 | 1.09 (0.72; 1.66) | 0.6857 |
| Missing |  |  | 1.00 (0.49; 2.06) | 0.9915 |
| **Self-reported Long Term Health condition** |  |  |  |  |
| No | Ref |  | Ref |  |
| Yes | 0.78 (0.57; 1.06) | 0.1086 | 0.74 (0.46; 1.20) | 0.6857 |
| Missing | 0.91 (0.61; 1.38) | 0.6658 | 0.86 (0.45; 1.63) | 0.6339 |
| **Clinical measures** |  |  |  |  |
| PHQ-9 <15 | Ref |  | **Ref** |  |
| PHQ-9>=15 | 1.31 (1.00; 1.72) | 0.0486 | **1.86 (1.24; 2.79)** | **0.0025** |
| GAD-7 <10 | Ref |  | **Ref** |  |
| GAD-7 >= 10 | 2.16 (1.59; 2.93) | <.0001 | **1.80 (1.15; 2.83)** | **0.0104** |
| **Waiting times** |  |  |  |  |
| <6 weeks referral to assessment (weeks) | Ref |  | **Ref** |  |
| >=6 weeks referral to assessment (weeks) | 0.74 (0.50; 1.07) | 0.1080 | **0.40 (0.23; 0.71)** | **0.0015** |
| <18 weeks referral to treatment (weeks) | Ref |  | Ref |  |
| >=18 weeks referral to treatment (weeks) | 0.94 (0.69; 1.30) | 0.7269 | 1.10 (1.24; 2.79) | 0.6736 |
| **Number of sessions** |  |  |  |  |
| 5 or more low intensity sessions | 2.03 (1.40; 2.96) | <0.0002 | **1.86 (1.24; 2.79)** | **0.0025** |
| 5 or more high intensity sessions | 1.85 (1.37; 2.51) | <.0072 | **1.80 (1.15; 2.83)** | **0.0104** |

**Table 7.4: Aim 3 – Reliable improvement for people living with an atypical dementia, compared to people with a typical dementia or no dementia**

|  | **Atypical Dementia**  **Vs Typical Dementia** |  | **Atypical Dementia**  **Vs No Dementia** |  |
| --- | --- | --- | --- | --- |
|  | **Odds Ratio (95%CI)** | **p-value** | **Odds Ratio (95%CI)** | **p-value** |
|  | **BROAD DEFINITION**  **N=2310** |  | **BROAD DEFINITION**  **N=1 881 510** |  |
| Model 1: Unadjusted | 1.26 (1.06; 1.50) | 0.0084 | 0.81 (0.72; 0.92) | 0.0008 |
| Model 2: Fully adjusted | 1.24 (1.04; 1.50) | 0.0167 | 0.77 (0.68; 0.87) | <.0001 |
| Model 3: Matched cohort, fully adjusted |  |  | N=2298  Match found for 1149 observations  0.71 (0.59; 0.86) | 0.0003 |

**Table 7.5: Aim 4: Reasons for discharge : Do reasons for discharge people differ compared to people with more typical dementia or without dementia?**

|  | **Atypical Dementia**  **Vs Typical Dementia** |  | **Atypical Dementia**  **Vs No Dementia** |  |
| --- | --- | --- | --- | --- |
|  | **Broad definition (inc G31.9)** |  | **Broad definition (inc G31.9)** |  |
|  | **RRR (95%CI)** | **p-value** | **RRR (95%CI)** | **p-value** |
| Multinomial logistic regression, adjusted as in Model 2 |  |  |  |  |
| Completed (ref) | Ref |  | Ref |  |
| Dropout or Declined | 0.90 (0.72; 1.12) | 0.3549 | 1.04 (0.89; 1.21) | 0.6519 |
| Referred on or Service not suitable | 0.77 (0.55; 1.07) | 0.1199 | 1.57 (1.25; 1.99) | 0.0001 |
| Missing | 0.76 (0.53; 1.09) | 0.1404 | 0.94 (0.71; 1.26) | 0.6941 |
|  |  |  |  |  |
| Multinomial logistic regression, adjusted as in Model 3, matched cohort | Not applicable |  |  |  |
| Completed (ref) |  |  | Ref |  |
| Dropout or Declined |  |  | 1.20 (0.95; 1.50) | 0.1356 |
| Referred on or Service not suitable |  |  | 1.48 (1.02; 2.12) | 0.0367 |
| Missing |  |  | 0.87 (0.58; 1.32) | 0.5161 |
|  |  |  |  |  |
| RRR = relative risk ratio | | | | |

# Supplementary analyses – Factors associated with reliable recovery and reliable deterioration for people with atypical dementia, multivariable logistic regression

**Table 8.1: Aim 2 – Factors associated with reliable recovery and reliable deterioration for people with atypical dementia, multivariable logistic regression**

|  | **Reliable Recovery** | | R**eliable Deterioration** | |
| --- | --- | --- | --- | --- |
|  | **N=523**  OR (95% CI) | p-value | **N=523**  OR (95% CI) | p-value |
| **Age Category** |  |  |  |  |
| <65 | Ref |  | Ref |  |
| 65+ | 1.35 (0.83; 2.21) | 0.2246 | **0.70 (0.30; 1.60)** | **0.3966** |
| **Ethnicity** |  |  |  |  |
| White | Ref |  | Ref |  |
| Asian | 0.29 (0.06; 1.39) | 0.1210 | 2.72 (0.52; 14.27) | 0.2366 |
| Black | 1.12 (0.30; 4.21) | 0.8671 | 1.11 (0.13; 9.87) | 0.9225 |
| Missing/Other | 0.69 (0.38; 1.23) | 0.2049 | 1.55 (0.61; 3.96) | 0.3600 |
| **Gender** |  |  |  |  |
| Male | Ref |  | Ref |  |
| Female | 0.88 (0.60; 1.28) | 0.5002 | 1.47 (0.74; 2.92) | 0.2696 |
| **IMD quintile** |  |  |  |  |
| 2-5 (Least deprived) | Ref |  | Ref |  |
| 1 (Most deprived) | 0.63 (0.39; 1.01) | 0.0573 | **0.83 (0.35; 1.95)** | **0.6650** |
| Missing | 0.67 (0.23; 1.94) | 0.4607 | **1.23 (0.25; 6.05)** | **0.7975** |
| **Taking Psychotropic medication** |  |  |  |  |
| No | Ref |  | Ref |  |
| Yes | 0.97 (0.65; 1.46) | 0.8828 | 1.61 (0.75; 3.48) | 0.2220 |
| Missing | 0.94 (0.45; 1.96) | 0.8726 | 1.46 (0.43; 4.92) | 0.5388 |
| **Self-reported Long Term Health condition** |  |  |  |  |
| No | Ref |  | Ref |  |
| Yes | 0.61 (0.39; 0.96) | 0.0306 | 1.02 (0.45; 2.34) | 0.9549 |
| Missing | 0.60 (0.32; 1.12) | 0.1069 | 1.19 (0.42; 3.39) | 0.7381 |
| **Clinical measures** |  |  |  |  |
| PHQ-9 <15 | Ref |  | **Ref** |  |
| PHQ-9>=15 | 0.82 (0.55; 1.21) | 0.3217 | 0.34 (0.17; 0.68) | 0.0022 |
| GAD-7 <10 | Ref |  | **Ref** |  |
| GAD-7 >= 10 | 0.91 (0.58; 1.43) | 0.6900 | 1.05 (0.49; 2.26) | 0.9047 |
| **Waiting times** |  |  |  |  |
| <6 weeks referral to assessment (weeks) | Ref |  | **Ref** |  |
| >=6 weeks referral to assessment (weeks) | 0.38 (0.21; 0.71) | 0.0024 | 0.76 (0.27; 2.11) | 0.5983 |
| <18 weeks referral to treatment (weeks) | Ref |  | Ref |  |
| >=18 weeks referral to treatment (weeks) | 1.06 (0.68; 1.66) | 0.7969 | 1.20 (0.57; 2.50) | 0.6302 |
| **Number of sessions** |  |  |  |  |
| 5 or more low intensity sessions | 1.72 (1.04; 2.86) | 0.0379 | 0.39 (0.11; 1.31) | 0.1268 |
| 5 or more high intensity sessions | 1.96 (1.31; 2.94) | 0.0011 | 1.01 (0.50; 2.07) | 0.9667 |

.

References

[1] National Collaborating Centre for Mental Health. The Improving Access to Psychological Therapies Manual. 2021.

[2] NHS Digital. Hospital Episode Statistics (HES). 2021.

[3] NHS Digital. Mental Health Services Data Set. 2021.

[4] NHS Digital. Linked HES-ONS mortality data. 2020.

[5] National Collaborating Centre for Mental Health. The Improving Access to Psychological Therapies Manual. National Collaborating Centre for Mental Health; 2021.

[6] Buckman JEJ, Saunders R, Stott J, Arundell LL, O'Driscoll C, Davies MR, et al. Role of age, gender and marital status in prognosis for adults with depression: An individual patient data meta-analysis. Epidemiol Psychiatr Sci. 2021;30:e42.

[7] Buckman JEJ, Saunders R, Stott J, Cohen ZD, Arundell L-L, Eley TC, et al. Socioeconomic Indicators of Treatment Prognosis for Adults With Depression: A Systematic Review and Individual Patient Data Meta-analysis. JAMA Psychiatry. 2022;79:406-16.

[8] Buckman JEJ, Stott J, Main N, Antonie DM, Singh S, Naqvi SA, et al. Understanding the psychological therapy treatment outcomes for young adults who are not in education, employment, or training (NEET), moderators of outcomes, and what might be done to improve them. Psychological Medicine. 2021:1-12.

[9] Saunders R, Buckman JEJ, Stott J, Leibowitz J, Aguirre E, John A, et al. Older adults respond better to psychological therapy than working-age adults: evidence from a large sample of mental health service attendees. Journal of Affective Disorders. 2021;294:85-93.

[10] Saunders R, Cape J, Leibowitz J, Aguirre E, Jena R, Cirkovic M, et al. Improvement in IAPT outcomes over time: are they driven by changes in clinical practice? Cogn Behav Therap. 2020;13:e16.

[11] Langworthy B, Wu Y, Wang M. An overview of propensity score matching methods for clustered data. Statistical Methods in Medical Research. 2022;32:641-55.
